# The importance of professional values from nursing students’ perspective

**DOI:** 10.1186/s12912-019-0351-1

**Published:** 2019-07-05

**Authors:** Batool Poorchangizi, Fariba Borhani, Abbas Abbaszadeh, Moghaddameh Mirzaee, Jamileh Farokhzadian

**Affiliations:** 1 ICU, Shahid Bahonar Hospital, Kerman University of Medical Sciences, Kerman, Iran; 2 Department of Nursing Ethics, Medical Ethics and law Research Center, Shahid Beheshti University of Medical Sciences, Tehran, Iran; 3 Bam University of Medical Sciences, Bam, Iran; 4 Department of Biostatistics and Epidemiology, School of Public Health, Kerman University of Medical Sciences, Kerman, Iran; 5 Nursing Research Center, Kerman University of Medical Sciences, PO Box: 7716913555, Kerman, Iran

**Keywords:** Nursing education, Professional values, Professional ethics, Nursing students

## Abstract

**Background:**

Professional values of nursing students may be changed considerably by curricula. This highlights the importance of the integration of professional values into nursing students’ curricula. The present study aimed to investigate the importance of professional values from nursing students’ perspective.

**Methods:**

This cross-sectional study was conducted at the Kerman University of Medical Sciences, Iran. Data were gathered by using a two-section questionnaire consisting of demographic data and Nursing Professional Values Scale-Revised (NPVS-R). By using the stratified random sampling method, 100 nursing students were included in the study.

**Results:**

Results showed that the mean score of the students’ professional values was at high level of importance (101.79 **±** 12.42). The most important values identified by the students were “maintaining confidentiality of patients” and “safeguarding patients’ right to privacy”. The values with less importance to the students were “participating in public policy decisions affecting distribution of resources” and “participating in peer review”. The professional value score had a statistically significant relationship with the students’ grade point average (*P* < 0.05).

**Conclusions:**

In light of the low importance of some values for nursing students, additional strategies may be necessary to comprehensively institutionalize professional values in nursing students.

## Introduction

Values are goals and beliefs that establish a behavior and provide a basis for decision making [1]. In a profession, values are standards for action that are preferred by experts and professional groups and establish frameworks for evaluating behavior [2]. Nursing is a profession rooted in professional ethics and ethical values, and nursing performance is based on such values. Core values of nursing include altruism, autonomy, human dignity, integrity, honesty and social justice [3]. The core ethical values are generally shared within the global community, and they are a reflection of the human and spiritual approach to the nursing profession. However, the values in the care of patients are affected by cultural, social, economic, and religious conditions dominating the community, making it essential to identify such values in each country [4].

Professional values are demonstrated in ethical codes [5]. In fact, ethical codes clarify nursing profession practices, the quality of professional care, and professional norms [2]. Advances in technology and expansion of nursing roles have provoked complex ethical dilemmas for nurses. Such dilemmas, if not dealt with properly, negatively affect the ability of novice nurses to make clinical decisions [6]. With the ever-increasing number and complexity of ethical dilemmas in care settings, promotion of professional values has become more crucial in nursing education. The acquisition and internalization of values are at the center of promoting the nursing profession [2]. When values are internalized, they will become the standards in practice and guide behavior [7]. Values can be taught, modified and promoted directly or indirectly through education [8]. Each student enters the nursing school with a set of values that might be changed during the socialization process [9]. Purposeful integration of professional values in nursing education is essential to guaranteeing the future of nursing [10, 11].

One of the significant consequences of teaching ethics and professional values to students is increasing their capacity for autonomous ethical decision-making [12]. Nursing students acquire professional values initially through the teaching of their school educators and the socialization process. Professional socialization is the method of developing the values, beliefs, and behaviors of a profession [13]. In their study, Seda and Sleem reported a significant relationship between professional socialization of students and improvement of professional values [9]. Through professional socialization, which results in the complete acquisition and internalization of values, nursing students should acquire necessary skills and knowledge in cognitive, emotional, and practical dimensions. Presently, however, less attention is paid to the emotional dimension in the formation of values compared to the other two [14]. In order to develop a value system, individuals should reach the fourth or fifth level of learning of Bloom’s affective domain, i.e. organization and internalization of values. At this level, stabilization of values requires passage of time [15].

Studies have shown that education causes differences in the formation of professional values, and that nursing educators have significant influence on the stimulation of professional values [8, 14, 16, 17]. Wehrwein reported that education related to ethics was effective when students’ awareness of ethical issues increased along with the application of values in the workplace. In addition, the ability to make ethical decisions was reported to be stronger in students who had passed an ethics course compared to those who had not [18]. Therefore, nursing educators play a key role in determining the future way in which nurses grow professionally and are prepared to confront new, unavoidable challenges [9].

Professors and educators, both in clinical settings and at each stage of education, have the role of facilitator in developing students’ perception of the nursing profession and the nurse’s role. Students may increase their commitment to professional values directly through role playing and indirectly through observing behaviors related to professional values [14]. Nursing educators are effective role models because of their clinical skills, sense of responsibility, professional commitment, and personal characteristics such as kindness, flexibility, and honesty. Nursing educators enhance creative learning by encouraging critical thinking and decision-making, establishing a supportive learning environment, having technical and ethical knowledge, and providing opportunities for fair evaluation and feedback. Nursing educators should teach nursing students effective strategies to confront ethical dilemmas [12].

Students’ perspectives on professional values influence their approach to applying professional values in their future profession [14, 15, 19, 20]. Nursing educators need additional awareness of nursing students’ perspectives on importance of professional values as a basis to use more effective methods for applying professional values. Therefore, nursing educators are able to educate graduates who are ready for decision-making and can effectively deal with daily ethical challenges. Nursing educators’ and students’ awareness of professional nursing values is important for preparing nurses to provide care of patients in an ethical and professional manner [6]. Researchers have found insufficient information about nursing students’ professional values in Iran. Because of the potential impact of cultures and clinical environments on professional values, the present study aimed to examine the importance of professional values from nursing students’ perspective.

## Methods

### Study design and setting

This cross-sectional study was performed from February to May 2016 at the Razi Nursing and Midwifery School affiliated with the Kerman University of Medical Sciences (KUMS), in Kerman, Iran. This study is a part of a larger study. The results of the first part was published in previous study [21].

### Sample

The participants included all undergraduate nursing students who were studying at the time of data collection (*n* = 177). The sample size (*n* = 106) was determined based on the Cochran formula (*d* = 0.06, *p* = 0.05). Inclusion criteria were undergraduate nursing students in the fourth, sixth, and eighth semesters without official work experience in hospitals. Submitting an incomplete questionnaire was considered an exclusion criterion. The participants were selected using a stratified random sampling based on the proportion of students in each semester. Therefore, among the total of 50, 62, and 65 students in the three semesters, 30, 37, and 39 students were enrolled, respectively. Finally, of the remaining 106 students, 100 students completed the questionnaires, but six students did not return the questionnaires. Thus, the final sample consisted of 100 students (with the response rate of 94.34%).

### Instrument

A two-section questionnaire was used for data collection. The students’ demographic data including age, grade point average (GPA: 17–20 (level A), 13–16 (level B) and ≤ 12 (level C)), ethnicity, gender, marital status, economic status of family, educational semester, and participation in professional ethical training courses was collected by the first section. The second section was Weis and Schank’s Nursing Professional Values Scale-Revised (NPVS-R). The NPVS-R is a potentially useful instrument for measuring professional nursing values. In developing the professional values scale, Weis and Schank used the ANA Code of Ethics as well as the studies on nursing values and their promotion among nurses [2].

We used the Persian version of the NPVS-R in this study. The validity of the translated questionnaire was confirmed using face and content validity as well as expert opinion. Reliability of the NPVS-R was reported to be 0.91 using Cronbach’s alpha [22]. To establish reliability of the NPVS-R in Persian, a pilot study was conducted with 20 nursing students, which resulted in a Cronbach’s alpha coefficient of 0.90.

The NPVS-R includes 26 items with a Likert-scale format in five dimensions: 1) trust: 5 items, 2) justice: 3 items, 3) professionalism: 4 items, 4) activism: 5 items, and 5) caring: 9 items. The trust dimension reflects the nurse’s duty (the value of veracity) to patients. The justice dimension deals with patients as noted in statements reflecting equality and diversity issues. The professionalism dimension reflects the promotion of nursing competence, self-evaluation and reflection, and seeking professional growth. The activism dimension reflects participation in professional activities and solutions to professional problems. The caring dimension reflects respect for patients and protection of patient rights.

The participants specified the importance of each item on a Likert 5-point scale ranging from 1 to 5 with 1 = not important, 2 = somewhat important, 3 = important, 4 = very important, and 5 = the most important. The possible range of scores is 26 to 130 [2]. In this study, the scores below 43, scores between 43 and 86, and those above 86 were considered low importance, moderate, and high importance, respectively. A higher score indicates that professional values are very important, and that nurses are more oriented toward stronger professional values.

### Data collection

The first researcher distributed the questionnaires among the participants and explained the study objectives. The researcher also explained to the participants how to fill out the questionnaires and asked them to specify the importance of professional values. In order to eliminate any ambiguity regarding questionnaire items, necessary explanations were provided. The researcher collected the questionnaires while maintaining anonymity and confidentiality of the data.

### Statistical analysis

Descriptive statistics (frequency, percentage, mean and standard deviation) and inferential statistics (independent samples, t- test, analysis of variance (ANOVA), Mann-Whitney, and Pearson’s correlation coefficient) were used. SPSS software version 19 was used for data analysis and level of significance was considered *p* ≤ 0.05.

### Ethical considerations

First, the study was approved by the ethics committee affiliated to the Kerman University of Medical Sciences (No code: 1394.238). Then, official permission for collecting data was obtained from the Razi Nursing and Midwifery School. Prior to distributing the questionnaire, the researcher guaranteed the confidentiality and anonymity of the questionnaires. The students’ informed consent was implied from returning completed questionnaires.

## Results

The results showed that the students’ mean age was) 21.9 ± 1.26 (and GPA was at B level (16.20 ± 1.20). Most of the students were female (75%), single (67%), and Iranian (97%). Around 37% of the students were in the eighth semester, and around 37.6% of the students had participated in professional ethical training courses (Table 1).

**Table 1:** Demographic data of the nursing students and its relationship with professional values

Variable		*N*	%	Mean ± SD	Test statistic	*p*
Gender	Female	75	75%	103.05 ± 1.26	*t* = 1.73	0.07
	Male	25	25%	98 ± 1.21		
Marital status	Married	33	33%	103.39 ± 1.25	*t* = 0.90	0.37
	Single	67	67%	101.66 ± 1.23		
Ethnicity	Persian	97	97%	101.6 ± 1.25	*Z* = 0.96	0.36
	Non Persian	3	3%	108 ± 7.93		
Semester	Fourth	29	29%	100.25 ± 1.25	*F* = 0.29	0.74
	Sixth	35	35%	102.49 ± 1.20		
	Eighth	37	37%	102.30 ± 1.28		
Participation in Professional ethical	Yes	156	62.4%	104.26 ± 1.18	*t* = 1.04	0.30
	No	94	37.6%	99.77 ± 1.17		

The high mean score of the professional values of the nursing students indicated high awareness and perception of the importance of professional values from the students’ perspective. The most important values as identified by higher mean scores were respectively as follows: “maintaining confidentiality of patients”, “safeguarding patients’ right to privacy”, “assuming responsibility for meeting health needs of the culturally diverse population”, and “maintaining competency in area of practice”. The values with lower mean scores were “participating in public policy decisions affecting distribution of resources”, “participating in peer review”, “recognizing role of professional nursing associations in shaping healthcare policy” and “participating in nursing research and/or implementing research findings appropriate to practice”, respectively (Table 2).

**Table 2:** Importance of professional values from the nursing students’ perspective

Dimension	Item	Rank	Mean	SD
Trust	Engage in ongoing self- evaluation	17	3.85	0.91
	Request consultation/collaboration when unable to meet patient needs	15	3.93	0.95
	Seek additional education to update knowledge and skills	6	4.25	0.83
	Accept responsibility and accountability for own practice	5	4.26	0.87
	Maintain competency in area of practice	4	4.31	0.76
Justice	Protect health and safety of the public	18	3.74	0.97
	Promote equitable access to nursing and healthcare	16	3.91	0.94
	Assume responsibility for meeting health needs of culturally diverse population	3	4.36	0.82
Professionalism	Participate in peer review	25	3.24	0.99
	Establish standards as a guide for nursing practice	21	3.66	0.1
	Promote and maintain standards where planned learning activities for students take place	11	4.00	0.91
	Initiate actions to improve environments of practice	8	4.07	0.79
Activism	Participate in public policy decisions affecting distribution of Resources	26	3.23	1.09
	Advance the profession through active involvement in health-related activities	14	3.95	0.90
	Recognize role of professional nursing associations in shaping healthcare policy	24	3.44	0.86
	Participate in nursing research and/or implement research findings appropriate to practice	23	3.50	0.93
	Participate in activities of professional nursing associations	22	3.56	1.01
Caring	Protect moral and legal rights of patients	7	4.22	0.74
	Refuse to participate in care if in ethical opposition to own professional values	13	3.96	0.97
	Act as a patient advocate	12	3.99	0.92
	Provide care without prejudice to patients of varying Lifestyles	10	4.05	0.94
	Safeguard patient’s right to privacy	2	4.43	0.70
	Confront practitioners with questionable or inappropriate Practice	20	3.68	1.03
	Protect rights of participants in research	19	3.68	0.93
	Practice guided by principles of fidelity and respect for Person	9	4.06	0.85
	Maintain confidentiality of patient	1	4.46	0.75
	Total		101.79	12.42

Adapted from Weis & Schank [2] and Poorchangizi et al. [21]

The results of the Pearson’s correlation coefficient test indicated that there was no significant relationship between professional values and age (*r* = 0.03, *p* = 0.47), while there was a significant relationship between professional values and the GPA (*r* = 0.29, *p* = 0.003). This revealed that the students with higher GPA had higher scores in professional values. There was no significant difference in the students’ professional values based on different educational semesters (*F* = 0.29, *p* = 0.74). In addition, there were no significant differences in professional values based on the other demographic variables such as gender, marital status, ethnicity, and participation in professional ethical training courses *(p* > 0.05) (Table 1).

## Discussion

The present study was conducted to examine the importance of professional values from nursing students’ perspectives. The results showed a high total score with regard to the importance of professional values. These findings are in agreement with the findings of the studies conducted in the United States [15, 23], Taiwan [24], Korea [25], and Iran [21]. Results of these studies highlighted that instructors and nursing trainers were seen as role models by students.

In this study, the most important nursing professional values were “maintaining confidentiality of patients”, “safeguarding patients’ right to privacy”, “responsibility for meeting health needs of the culturally diverse population”, and “maintaining competency in area of practice”. The results of this study are in agreement with the results of the studies conducted by Lin et al. [19], Clark [15], Fisher [23], and Leners et al. [8], who identified these values as the most important values. One possible reason for the consistency between the results of this study and those of the other studies may be that these values are among the main values in the nursing profession and are closely associated with it. Leners et al. reported that maintaining competency in area of practice, accepting responsibility and accountability for own practice, and safeguarding patients’ right to privacy were values prioritized by students. Since these values are associated with the direct care of patients and given that students complete their clinical practices under supervision of nurses, students may learn the importance of these values through role modeling and application in clinical settings [8].

In this study, the least important values from the students’ perspective were “participating in public policy decisions affecting distribution of resources”, “participating in peer review”, “recognizing role of professional nursing associations in shaping healthcare policy”, and “participating in nursing research and/or implementing research findings appropriate to practice”*.* The results of this research are also in agreement with the results of the studies conducted by Lin et al. [19], Clark [15], Fisher [23], and Leners et al. [8]. A multitude of factors may have contributed to the lower importance placed on these values; some causes might be less information about the importance of these values in the development of the profession, low motivation, insufficient affirmation, and low encouragement by nursing educators.

The reason for the low importance placed on the values such as “participating in nursing research and/or implementing research findings appropriate to practice” might be the fact that nursing students do not acquire necessary skills (such as information literacy skills) to apply in evidence-based practices during their academic days [26, 27]. Another reason for the low importance of the above-mentioned values might be graduate education programs; undergraduate students focus on the rules of clinical practice because they are novices. As they become more competent and eventually experts, the ranking of the values is likely to change.

Concerning the lower importance of the “recognizing role of professional nursing associations in shaping healthcare policy” value, Esmaeili et al. reported the following items as causes for reduction of participation in such associations: long working hours, lack of awareness about the associations’ objectives and activities, insufficiency of time, and lack of support from hospitals to play active roles in associations. Moreover, the inactivity of members in such associations and the weak relationship among these associations were other barriers confronted by such associations in Iran [28]. In addition, one other reason might be that nursing educators themselves do not participate in professional nursing associations because of high workloads and limited time. Professional nursing associations play major roles in promoting nursing authority and professional identity. Consequently, understanding and valuing the importance of participation in professional associations may require emphasis as an important professional value.

Regarding the low importance of values such as “participating in peer review” and “participating in public policy decisions affecting distribution of resources”, it can be mentioned that these activities are part of the manager’s duties, and that the nurses are not involved in peer evaluation and policy decisions.

In this study, a significant relationship was found between the GPA and scores of professional values. Students with high GPA Probably have the necessary scientific competency in their professional performance, which may result in giving higher importance to professional values as a significant index of professional competence. Lechner et al. emphasized that academic environments appear to elicit and reinforce values such as developing one’s capacities and pursuing one’s interests [29].

In this study, although no significant difference was found between educational semester and the students’ scores of professional values, the highest score was related to the sixth semester whereas the lowest score was related to the fourth semester. The studies conducted by Rassin [16] and Clark [15] had results similar to those of this study, with no difference found between total scores of professional values of students in different semesters of their nursing education. However, several studies [8, 14, 25] found significant differences between total scores of professional values of students in different semesters. It is difficult to compare these differences due to the use of different instruments to measure professional values, differences in nursing education curricula and environments, and differences in study designs. In line with the results found on the association between academic year and education with professional values, researchers reported in several studies that education had a positive effect on professional values, and nursing students’ education experience increased total scores of professional values in a positive direction from entry into school until graduation [8, 14, 19]. In their study, Weis and Shank concluded that higher focus on curricula of junior and senior students could change some professional values, indicating that time spent in school was associated with change in values [30].

The study had three limitations. First, the assessment of the students’ perspectives on professional values was limited only in the school affiliated with the KUMS in southern Iran, which may limit generalization of these findings. The second limitation was the translation of the NPVS-R into Persian. Cultural and language differences may have affected the meaning of the terms literally and in the context of nursing education in Iran. Third, this study did not assess how the students learned professional values. Similarly, we did not know to what extent students had these values prior to entering nursing education and we did not collect information on these two items; thus, we did not highly emphasize the role of nurse educators in this study. It is suggested that further studies with more accurate instruments are conducted in other nursing schools with different cultural and environmental conditions might lead to comprehensive strategies for internalizing professional values of nursing students.

### Implications for nursing

Nursing educators can primarily facilitate professional values by urging students to participate both in research studies on the topic and in nursing education. Periodic classes and seminars about professionalism should be presented by clinical tutors and school educators, who play important roles as behavioral models for their students. It is also recommended to conduct studies to investigate the impact of educational environments and university educators as role models for students on advancement of professional values in students.

## Conclusions

The study showed high mean total of professional values from the nursing students’ perspective. However, some professional values such as participating in public policy decisions and participating in nursing researches were less important. This shows low awareness about these values or educators’ insufficient emphasis on them, time limitations to promote these values, and negative attitudes of students toward these values. As future nurses, nursing students should be able to apply professional values in making decisions when confronted with the emerging ethical challenges in the healthcare area. This preparation should be provided for students by educators and professors during their professional socialization process in schools. The findings suggest that many of the values were similarly important in other countries, which can be a reflection of the globalization process in the nursing profession and the presence of professional values at the root of the discipline. However, strategies should be developed to improve weaknesses of nursing students in the professional values adapted to cultural, social, and religious conditions prevailing in the societies, faculties, schools, and hospitals.

## Data Availability

The datasets generated and analyzed during the current study are not publicly available because this study is part of a larger study. This datasets are available from the corresponding author on reasonable request.

## Abbreviations

GPA: Grade point average
NPVS-R: Nursing Professional Values Scale-Revised
